# Trends in the Attack Rates, Incidence, and Mortality of Stroke during 1986–2012: Data of Kaunas (Lithuania) Stroke Registry

**DOI:** 10.1371/journal.pone.0153942

**Published:** 2016-04-28

**Authors:** Ricardas Radisauskas, Vilija Malinauskiene, Egle Milinaviciene, Daina Kranciukaite-Butylkiniene, Abdonas Tamosiunas, Gailute Bernotiene, Dalia Luksiene, Zemyna Milasauskiene, Diana Sopagiene, Daiva Rastenyte

**Affiliations:** 1 Institute of Cardiology, Lithuanian University of Health Sciences, Kaunas, Lithuania; 2 Department of Environmental and Occupational Medicine, Lithuanian University of Health Sciences, Kaunas, Lithuania; 3 Department of Rehabilitation, Lithuanian University of Health Sciences, Kaunas, Lithuania; 4 Department of Family Medicine, Lithuanian University of Health Sciences, Kaunas, Lithuania; 5 Department of Preventive Medicine, Lithuanian University of Health Sciences, Kaunas, Lithuania; 6 Department of Public Health, Klaipeda University, Klaipeda, Lithuania; 7 Department of Neurology, Lithuanian University of Health Sciences, Kaunas, Lithuania; National Cardiovascular Center Hospital, JAPAN

## Abstract

**Background:**

There is a lack of reliable epidemiological data on longitudinal trends in stroke attack rates, incidence, and mortality in the countries of the Baltic region.

**Aims:**

The aim of the present study was to explore the longitudinal trends of stroke in middle-aged urban population of Lithuania during the period of 1986 through 2012.

**Methods:**

All stroke events in the studied population were ascertained and validated according to the standardized criteria outlined by the WHO MONICA Project. The study included all patients in Kaunas (Lithuania) city aged 25 to 64 years who experienced a stroke between 1986 and 2012. Estimates of time-trends of the annual percentage change in stroke attack rates, incidence of stroke, and mortality from this condition were made by applying the Joinpoint regression analysis.

**Results:**

During the study period, 9,992 stroke events were registered. The overall proportion of recurrent events was 25.7%. Overall, 18.9% of the events (20.0% in men, and 17.4% in women) were fatal within 28 days. During the period of 1986 to 2012, a flat trend in the incidence of stroke was observed among both male and female middle-aged inhabitants of Kaunas city, while attack rates were increasing due to the increase in recurrent strokes. Both mortality and 28-day case fatality of stroke declined significantly over the study period in both sexes.

**Conclusions:**

An increase both in the incidence and recurrence of stroke among middle-aged men residing in Kaunas city and in the recurrence of stroke among women denotes the inefficiency of measures applied both for primary and secondary prevention of stroke in Lithuania. The revision of current prevention strategies and the introduction of new ones are of paramount importance in order to fight the epidemic of stroke.

## Introduction

Even though divergent patterns of stroke epidemiology in different socioeconomic regions of the world exist as from 1990 to 2010, the age-standardized incidence of stroke significantly decreased by 12% in high-income countries, and increased by 12% in low-income and middle-income countries–albeit non-significantly so [1]. Mortality rates decreased significantly in both high-income (37%) and low-income and middle-income countries (20%) [1–2]. Although age-standardized mortality rates for ischemic and hemorrhagic stroke have decreased over the past two decades, the absolute number of people who have these stroke types–as well as the number of related deaths–is increasing annually, with most of the burden falling on low-income and middle-income countries [3].

In general, in recent decades, the trends in stroke incidence and mortality rates have decreased for high-income countries such as the USA, Canada, the United Kingdom, Sweden, Spain, etc. [4–8]. The decline in stroke mortality over the past decades–a major improvement in the population’s health observed for both sexes and all race and age groups–has resulted from reduced stroke incidence and lower mortality rates [4], and the decrease in the percentage of stroke hospitalizations resulting in death observed over the last decade likely reflects advancements in acute stroke care [9]. A higher burden of stroke-related mortality is estimated in North Asia, Eastern Europe, Central Africa, and the South Pacific [10].

In Lithuania, time trends in stroke mortality were investigated on the basis of data obtained during the WHO MONICA Project; and a significant decline in stroke mortality rates was observed in middle-aged male and female population of Kaunas city during the period of 1986 to 2002, while the incidence of stroke demonstrated no trends in men and an increasing one in women [11].

This study reports on the time trends of attack rates, incidence, and mortality from stroke according to the population-based stroke register data for the period of 1986 through 2012.

## Material and Methods

### Ethics statement

The study protocol was approved by the Regional Biomedical Research Ethics Committee at the Lithuanian University of Health Sciences. All patient records/information were anonymized and de-identified prior to the analysis.

### Study sample

The source of the population data was the Central Statistical Department of Kaunas city (Lithuania), which yearly publishes reports on the population size in five-year age groups. The size of the population aged 25–64 years was 95,437 men and 117,059 women in 1986, and 74,303 men and 90,853 women in 2012. Stroke registration methods strictly followed the protocol and quality control procedures laid down in the WHO MONICA project [12]. The “cold pursuit” technique (i.e. retrospective data collection) was used to identify stroke events [12]. Multiple sources of information (hospital discharge records, records of domiciliary care of outpatient departments, autopsy, and medico-legal records) were used for case ascertainment—including death certificates for permanent residents of Kaunas city.

These sources were reviewed every three months—except for death certificates, which were reviewed every month. All suspected acute stroke events were recorded on special forms translated from the Stroke Events Registration Form of the WHO MONICA project [12]. According to the WHO MONICA protocol [12], stroke was defined as “rapidly developed clinical signs of focal (or global) disturbance of cerebral function lasting more than 24 hours (unless interrupted by surgery or death) with no apparent cause other than a vascular origin”. Global symptoms apply to patients with coma or subarachnoid hemorrhage (SAH) without focal neurological signs.

Every stroke event had to have its apparent onset within the study period, and had to occur more than 28 days after any previously recorded stroke event in the same subject. Multiple stroke attacks occurring within 28 days from the onset of the symptoms of the first attack were considered as one event. A stroke event was defined as fatal if death occurred within the first 28 days from the onset. If the patient was alive after 28 days from the onset of the attack, the stroke was classified as non-fatal. All patients residing permanently in Kaunas city and suspected of having died from stroke or having had a non-fatal acute stroke were registered. These events were classified into one of the following diagnostic categories: definite stroke, definite stroke associated with definite myocardial infarction, no stroke event, and an unclassifiable event. The latter category was mainly used in fatal cases without autopsy or computed tomography (CT) performed, where acute cerebrovascular disease was indicated as the cause of death on the death certificate, but the clinical information was too limited to classify the event as a definite stroke. Unclassifiable stroke accounted for 2.4% of the events. There were 206 cases (2.0% of all stroke events) with a diagnostic category of “no stroke”. Definite stroke events (fatal and non-fatal) and unclassifiable events (fatal and non-fatal) were used in the analysis.

The codes for the specific types of stroke were confirmed by specific diagnostic examinations. For subarachnoid hemorrhage (SAH), autopsy, brain CT, or sampling of cerebrospinal fluid containing blood were required to determinate the diagnosis, while for intracerebral hemorrhage (ICH), the diagnosis had to be confirmed by CT or by autopsy. Ischemic stroke (IS) was diagnosed when CT and/or autopsy could verify the infarction and/or exclude hemorrhage and non-vascular disease. If it was impossible to assign a definite stroke event to one of these types, the type of stroke had to be coded as “an acute, but ill-defined cerebrovascular disease”. These cases were assigned to the type of IS in specific stroke type analyses.

In this study, the term “incidence rate” referred to the rate of first-ever events, i.e. those that occurred without any evidence of a previous stroke. The term “attack rate” referred to the rate of all episodes of stroke (first and recurrent events). Mortality from stroke was calculated for all events that were fatal within 28 days from the onset of the symptoms. The term “case-fatality rate” referred to the proportion of stroke patients who died within 28 days from onset of the symptoms of acute stroke.

Throughout the study period, the same methods for the identification of stroke cases, the same diagnostic criteria of stroke, and the same quality assurance procedures were applied in order to assure comparability of the data [13].

### Statistical analysis

All the incidence, attack, and mortality rates presented were directly standardized by five-year groups to the world standard population [14, 15]. The 95% confidence intervals (CI) were calculated assuming the Poisson distribution within the age strata. The case-fatality rate was age-adjusted by means of weights proposed for use in the WHO MONICA Project [15]. Changes in rates over the study period were analyzed using the Joinpoint regression program [16]. For all rates of interest, four joinpoint regression models were computed: zero (0) joinpoints were assumed in the first model, and three (3)—in the fourth one. The true number of joinpoints (from 0 to 3) was determined by means of the permutation test. The estimated regression coefficient (beta) multiplied by 100 was given as the estimated annual percentage change (APC). The 95% CI of the APC was obtained in the usual manner from the standard error of the regression coefficient. Differences in rates at the level of p<0.05, using a two-tailed test, were reported as statistically significant.

## Results

During the 27-year study period, 9,992 stroke events were registered among 25–64 year-old males and females residing in Kaunas city. The overall proportion of recurrent events was 25.7%. Overall, 1,889 (18.9%) events (1,132 (20.0%) in men, and 757 (17.4%)—in women) were fatal within 28 days. In men, 53.7% of all deaths from stroke were due to IS, 33.5%—from ICH, and 11.3%—from SAH. In women, the majority of all deaths from stroke (58.3%) were due to hemorrhagic strokes (ICH and SAH taken together). Deaths from ischemic strokes comprised 40.2%.The numbers of the registered stroke events and the proportions of CT scans and autopsies performed in male and female residents of Kaunas city by age groups and three 9-year time periods are presented in Table 1. The proportion of CT scans performed increased significantly from 1986–1994 to 2004–2012, while the proportion of autopsies decreased. At the same time, a clear disparity in the use of CT scans with increasing age was observed in 1986–1994, which completely disappeared in the last analyzed period.

**Table 1:** Numbers of registered stroke events and proportions of computed tomography and autopsy performed among 25–64 year-old male and female residents of Kaunas city by age-group and 9-year periods during 1986–2012.

Years/	All	First-	Recurrent	Fatal	stroke	events		CT	Autopsy
Age	Strokes	ever	stroke					%	rate
	Events	stroke	events	All	IS	ICH	SAH		%
		events	n	n	n	n	n		
	N	n							
**Men**									
**1986–1994**	1954	1475	479	513	325	136	49	19.5	68.2
**25–34**	45	42	3	15	4	6	5	46.7	86.7
**35–44**	166	145	21	54	19	22	13	28.3	81.5
**45–54**	623	482	141	136	74	43	16	22.0	77.2
**55–64**	1120	806	314	308	228	65	15	15.8	61.0
**1995–2003**	1857	1300	557	340	155	135	43	49.5	63.5
**25–34**	44	44	0	10	2	5	3	86.4	90.0
**35–44**	133	113	20	33	4	16	13	69.9	78.8
**45–54**	521	385	136	83	32	38	10	52.4	69.9
**55–64**	1159	758	401	214	117	76	17	44.5	57.5
**2004–2012**	1841	1339	502	279	128	108	36	92.9	31.2
**25–34**	41	40	1	9	0	5	3	73.2	44.4
**35–44**	149	117	32	18	3	10	4	90.6	55.6
**45–54**	515	410	105	86	27	41	17	93.4	33.7
**55–64**	1136	772	364	166	98	52	12	93.8	26.5
**Women**									
**1986–1994**	1289	1021	268	328	155	127	44	20.6	73.5
**25–34**	33	27	6	6	0	2	3	63.6	100.0
**35–44**	111	86	25	19	6	9	4	32.4	84.2
**45–54**	403	327	76	87	24	49	13	25.3	78.2
**55–64**	742	581	161	216	125	67	24	14.3	69.9
**1995–2003**	1642	1221	421	250	92	105	51	38.9	67.6
**25–34**	28	26	2	8	2	1	5	20.6	87.5
**35–44**	107	85	22	20	2	10	8	89.3	85.0
**45–54**	498	385	113	62	12	35	15	57.9	77.4
**55–64**	1009	725	284	160	76	59	23	37.8	60.6
**2004–2012**	1409	1073	336	179	57	73	41	91.6	29.6
**25–34**	18	17	1	2	1	0	1	94.4	50.0
**35–44**	106	89	17	17	0	8	7	85.8	47.1
**45–54**	468	363	105	50	14	21	15	89.3	36.0
**55–64**	817	604	213	110	42	44	18	93.5	23.6

CT–Computed tomography; IS–ischemic stroke (combined thrombotic, embolic, and unspecified type of stroke); ICH–intracerebral haemorrhage; SAH–subarachnoid haemorrhage, n–number of cases.

During the period of 1986 to 2012, the attack rates of stroke among middle-aged men and women residing in Kaunas city were increasing, although the trend was significant only among women (Table 2 and Table 3). The incline of stroke attack rates among men resulted from the increase in the rates of both incident and recurrent strokes—especially during 1997–2012 (Table 2). Mortality from all strokes was declining throughout the entire 27-year study period while some flattening of the trend was observed from 1997 onwards (Table 2). The 28-day case-fatality of stroke was constantly declining by 2.9% per year (Table 2).

**Table 2:** Trends in age-standardized stroke rates among 25–64 year male residents of Kaunas city during the period of 1986–2012 by Joinpoint regression analysis.

****Join-points (Years)****	****Time period****	****APC****	****95% CI****	****p value****
***Attack rate***				
0 joinpoint	1986–2012	+0.5	-0.03–1.0	0.07
1994	1986–1994	+3.3	0.8–6.0	<0.05
1997	1994–1997	-7.6	-26.5–16.0	0.5
	1997–2012	+1.5	0.5–2.5	<0.05
***Incidence***				
***of all strokes***				
0 joinpoint	1986–2012	+0.3	-0.3–0.9	0.3
1994	1986–1994	+2.5	-0.3–5.4	0.1
1997	1994–1997	-8.8	-29.3–17.6	0.4
	1997–2012	+1.9	0.8–3.0	<0.05
***Recurrence***				
***of all strokes***				
0 joinpoint	1986–2012	+1.0	0.3–1.7	<0.05
***Mortality from***				
***all strokes***				
0 joinpoint	1986–2012	-2.4	-3.5–-1.3	<0.05
1993	1986–1993	+6.5	-0.6–14.1	0.1
1997	1993–1997	-15.3	-34.5–9.6	0.2
	1997–2012	-0.5	-2.6–1.7	0.7
***Case fatality of***				
***all strokes***				
0 joinpoint	1986–2012	-2.9	-3.9 –-1.9	<0.05

APC–annual percent change; CI–confidence intervals.

**Table 3:** Trends in age-standardized stroke rates among 25–64 year-old female residents of Kaunas city during 1986–2012 by Joinpoint regression analysis.

****Join-points (Years)****	****Time period****	****APC****	****95% CI****	****p value****
***Attack rate***				
0 joinpoint	1986–2012	+0.9	0.2–1.5	<0.05
1996	1986–1996	+3.0	2.0–4.0	<0.05
2007	1996–2007	-3.2	-0.7 –-2.8	<0.05
2010	2007–2010	+13.6	-5.6–36.7	0.1
	2010–2012	-17.4	-31.3–-0.6	<0.05
***Incidence***				
***of all strokes***				
0 joinpoint	1986–2012	+0.6	-0.1–1.2	0.1
***Recurrence***				
***of all strokes***				
0 jpoinpoint	1986–2012	+1.8	0.5–3.2	<0.05
1995	1986–1995	+8.8	2.4–15.6	<0.05
	1995–2012	-0.8	-3.0–1.6	0.5
***Mortality from***				
***all strokes***				
0 joinpoints	1986–2012	-3.2	-4.5 –-1.7	<0.05
***Case-fatality of***				
***all strokes***				
0 joinpoint	1986–2012	-3.9	-5.2–-2.6	<0.05
1993	1986–1993	+4.0	-3.3–11.9	0.3
2000	1993–2000	-14.3	-21.9 –-6.0	<0.05
2003	2000–2003	+23.2	-28.7–112.8	0.4
	2003–2012	-7.7	-12.2–-3.0	<0.05

APC–annual percent change; CI–confidence intervals.

Among women, an increasing trend in attack rates of stroke could be solely attributed to the increase in the rates of recurrent strokes, as no changes were observed in the incidence of stroke (Table 3). Both mortality and 28-day case fatality rates declined significantly during the study period, while some inconsistency could be noticed in the trends of case fatality rates (Table 3).

**Fig 1:**
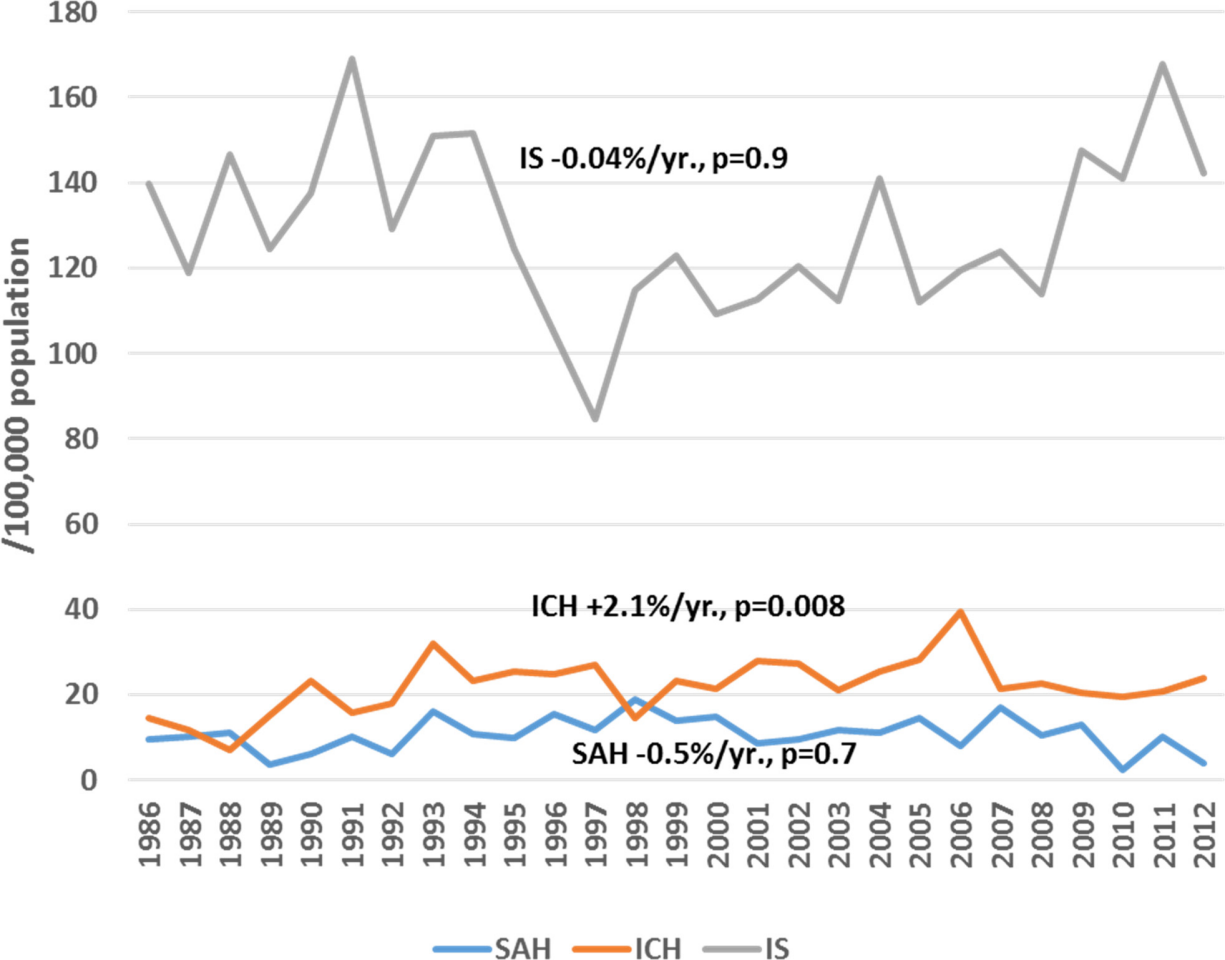
Trends in age-standardized incidence of stroke by types among 25–64 year male residents of Kaunas (Lithuania) during 1986 to 2012.

**Fig 2:**
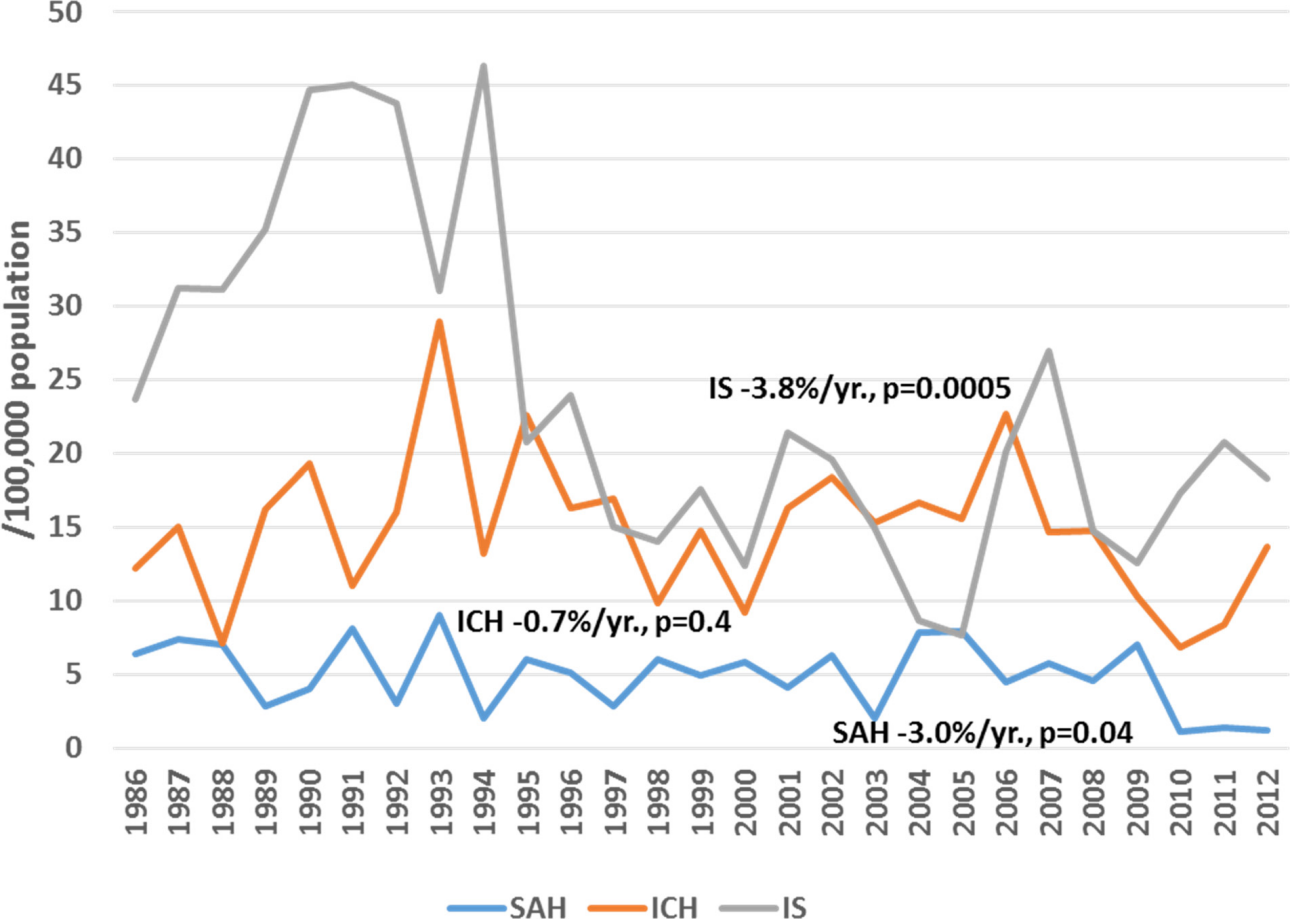
Trends in age-standardized mortality of stroke by types among 25–64 year male residents of Kaunas (Lithuania) during 1986 to 2012.

**Fig 3:**
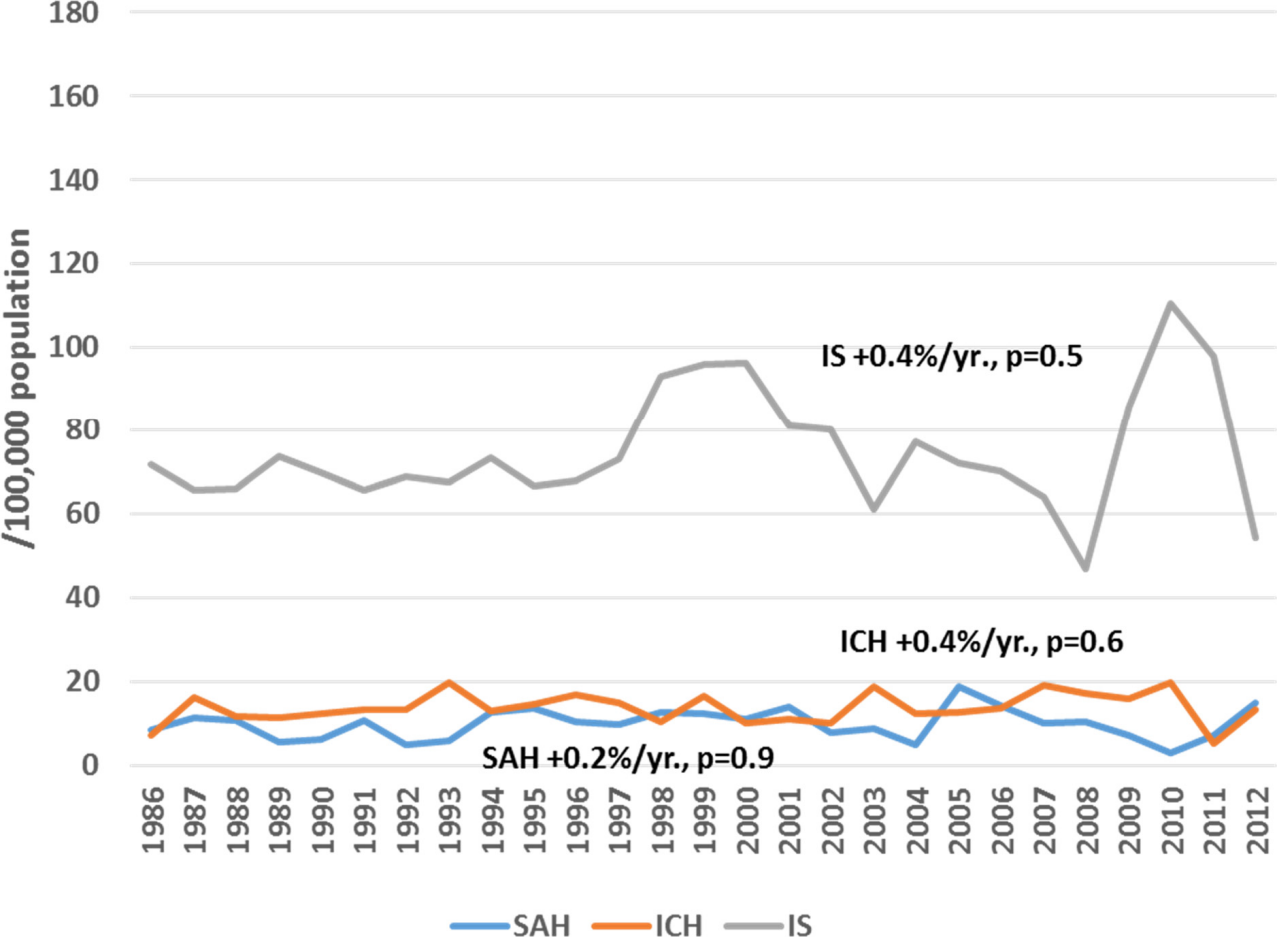
Trends in age-standardized incidence of stroke by types among 25–64 year female residents of Kaunas (Lithuania) during 1986 to 2012.

**Fig 4:**
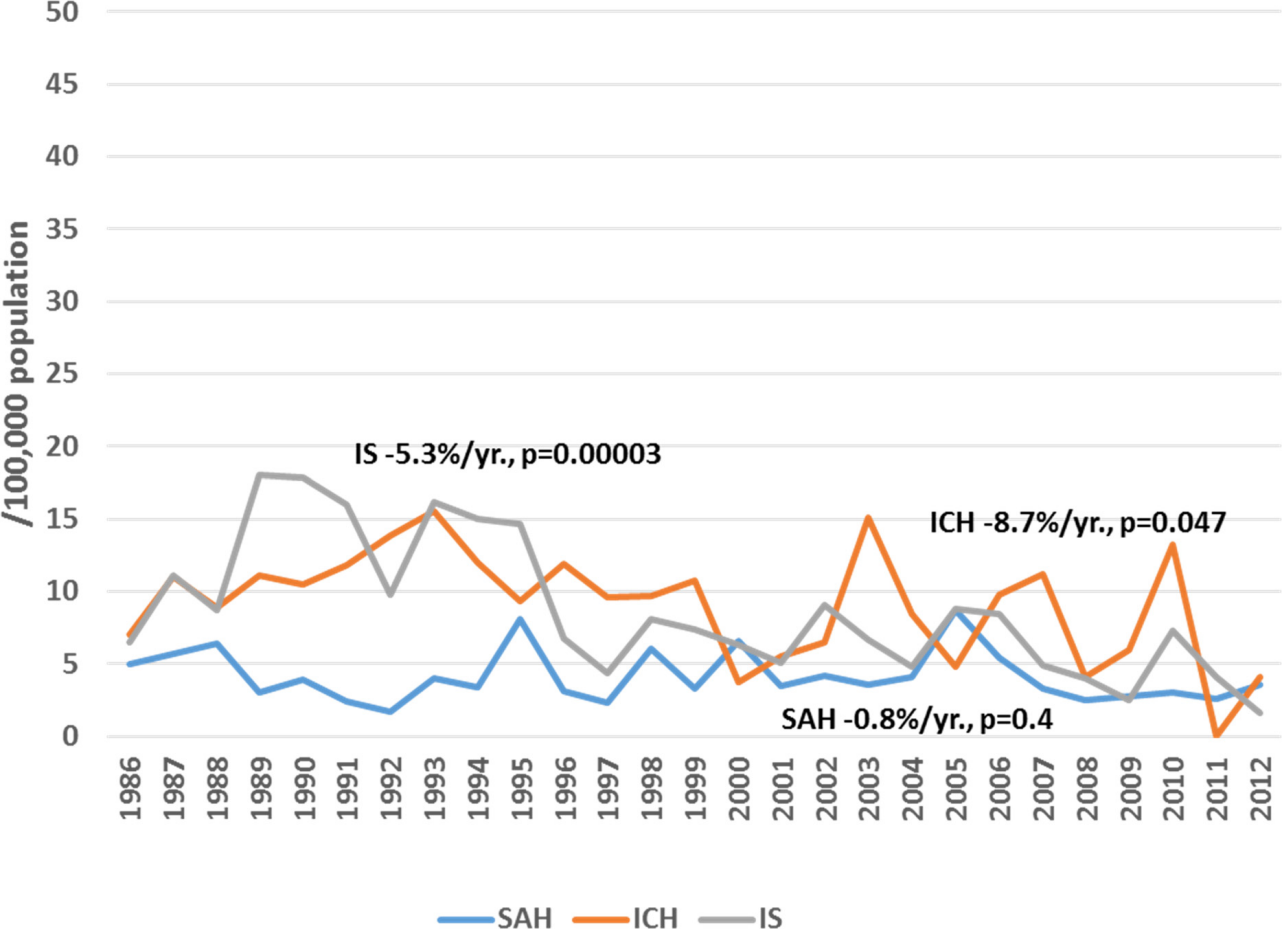
Trends in age-standardized mortality of stroke by types among 25–64 year female residents of Kaunas (Lithuania) during 1986 to 2012.

Among male residents of Kaunas city, a significant increasing trend in attack rates (+2.1%/year, p<0.05) and the incidence of ICH (+2.1%/year, p<0.05) was observed during the period of 1986 to 2012, while during 1997–2012, a significant increasing trend was observed in attack rates and the incidence of IS (by 2.0%/year and by 2.5%/year, p<0.05, respectively) (Fig 1). Also, the rates of recurrent IS were increasing by 0.9%/year (p<0.05) during 1986–2012. Conversely, declining trends were observed in mortality rates of both IS and SAH among middle-aged men residing in Kaunas city through the entire study period, while no significant changes could be seen in mortality rates from ICH (Fig 2). Among middle-aged female residents of Kaunas city, flat trends both in attack rates and the incidence of ICH (+0.4%/year, p = 0.6, and +0.4%/year, p = 0.6, respectively) were observed during the study period. Also, attack rates and the incidence of IS were without any significant changes during the period of 1986–2012 (+0.7%/year, p = 0.1 and +0.4%/year, p = 0.5) (Fig 3), while the rates of recurrent IS were increasing by 1.7%/year, p<0.05. Among women, a significant declining trend was observed in mortality from IS and haemorrhagic stroke, while mortality from SAH remained stable during the 27-year study period (Fig 4).

The case-fatality rates of IS, ICH, and of SAH were decreasing significantly both among men (by 4.0%/year, 2.8%/year, and by 2.8%/year, respectively) and women (by 6.0%/year, 7.9%/year, and by 2.0%/year, respectively) over the study period.

## Discussion

The main purpose of this study was to determine time trends in attack rate, incidence, and mortality from stroke in the middle-aged population of Kaunas city during the period of 1986 through 2012. No trends in the incidence of stroke, increasing trends in the rates of recurrent strokes, and declining trends in mortality and 28-day case-fatality of stroke were found both among men and women over the 27-year study period. Among men, however, an increase in the incidence of all strokes, IS, and hemorrhagic strokes was observed during the last 16 years. As compared to the previous findings on stroke trends in the same population published in 2006 [11], the later changes as well as a significant increase in stroke recurrences are amongst the most alarming ones.

A systematic review of the population-based studies demonstrated that over the past four decades, stroke incidence rates in high-income countries decreased by 42%, whereas in low- to middle-income countries, the stroke incidence rates increased, on the average, by 2.3 times [17]. More recent studies have shown a declining trend in stroke incidence in the United States [18] and Western and Northern European countries such as the United Kingdom [19], Scotland [20], and Spain [8] during the past decades. Meanwhile, studies from Northern and Southern Sweden [21, 22], and France [23] reported stable stroke incidence rates over the past 20 years. None of these studies reported divergent trends for men and women. A recently published study from the Netherlands indicated that stroke incidence rates decreased by 34% in men, but remained unchanged in women during the period of 1990 to 2008 [24]. The significant decrease in stroke incidence in high-income countries was largely due to a reduction in the incidence of cerebral infarction and ICH. In the present study, however, the increasing trend in the incidence of stroke observed among middle-aged men residing in Kaunas city resulted from the increase in the incidence of both IS and ICH, and corresponded to the trend observed in the incidence of acute myocardial infarction [25].

A lot of previous studies—including the recent ones from Oxfordshire and London (UK) and from Beijing, Shanghai, and Changsha (China) [17]—suggested that the implementation of preventive treatments and the reductions in risk factors at the population level contributed to a significant reduction in the incidence of stroke [26, 27, 28]. A significant decline in the incidence and attack rates of stroke was also linked to the use of medications that attenuate the risk of stroke [18]. Antihypertensive medications were shown to reduce the risk of stroke by approximately 32%, and statins—by approximately 21% [29, 30].

No reduction, but rather an increase in the main cerebrovascular risk factors could be noticed in the population of Kaunas city during the last few decades—especially among men [31]. For instance, during the period of 1986–1987 to 2006–2008, mean systolic blood pressure of middle-aged men residing in Kaunas city increased from 141.8 mmHg to 145.5 mmHg, as did prevalence of arterial hypertension (from 62.7% to 74.0%) and regular smoking (from 36.5% to 44.6%). All these changes were statistically significant (Tamosiunas A., personal communication). Although a significant increase in hypertension awareness among hypertensive male and female residents of Kaunas city (from 45.0% to 64.4%, and from 47.7% to 72.3%, respectively) and among treated hypertensive patients (from 55.4% to 68.3% in men, and from 65.6% to 86.2% in women) was observed during the period of 1983 to 2009 [32], the overall situation suggests poor control of hypertension. Also, a striking rise in the prevalence of regular smoking among female inhabitants of Kaunas city was observed during the period of 1986–1987 to 2006–2008 (from 2.6% to 15.7%) (Tamosiunas A., personal communication). These unfavorable changes in the risk factor profile could at least in part explain the unfavorable trends in stroke event rates observed in the present study.

Secondary prevention measures are of great importance when fighting stroke recurrence. In addition to an efficient control of the main cerebrovascular risk factors, the use of antiagregants or anticoagulants (in case of cardioembolic IS due to atrial fibrillation) has been proven as effective to prevent recurrent stroke [33]. Nevertheless, the reported prescription of oral anticoagulants for secondary stroke prevention in European countries was only about 50% [34]. The situation with anticoagulants for secondary prevention of stroke might be even worse in Lithuania, as only 37% of stroke patients with a history of atrial fibrillation were on anticoagulant therapy before the onset of stroke [35].

During the period of 1986 to 2012, mortality rates from stroke significantly declined both in male and female middle-aged inhabitants of Kaunas city. The decline in 28-day case-fatality rates explained 100% of the observed decline in mortality.

Declining stroke mortality rates among both males and females have recently been reported in the United Kingdom [19, 36, 37], Estonia [38], France [39], and the Netherlands [40]. During the period of 1980 to 2005, the sharpest decline (by more than >4% per year) in stroke mortality rates was observed in France followed by the United Kingdom, Norway, and Finland, were stroke mortality rates were declining by almost 3% per year [37], which corresponds to the findings of the present study.

While in most countries, the reported decline in stroke mortality over the last decades has resulted from reduced stroke incidence and case-fatality rates [4, 41], this is not the case in the present study, where decreasing mortality rates can be solely attributed to the decrease in case-fatality rates. As commonly acknowledged, the decline in case-fatality rates could be explained by less severe strokes or by improvements in stroke management. Keeping in mind an unfavorable risk profile of the population of Kaunas city as described above, the latter explanation seems to be most plausible. Already at the end of the 1990s, two stroke units were established in two major local hospitals of Kaunas city [11]. Although only about 26% of all stroke patients were admitted to a stroke unit in Kaunas in 2006 [35], the stroke management system has been constantly improving in Lithuania. All novel therapies—including thrombectomy—are now available for stroke patients in Lithuania, and the Ministry of Health is actively supporting the integration of all elements of stroke care at all levels of the healthcare system.

On the other hand, a declining mortality from stroke could be the result of a better detection of the less severe stroke events or because of improved diagnostics facilities [42, 43]. Nevertheless, this hardly could be the case in Kaunas city, since the usage of MRI in case of acute stroke is still quite rare (about 1.5% or even less) [35], and CT scan remains the diagnostic method of choice in case an acute cerebrovascular event is suspected. The definition of acute stroke used in the present study does not account for radiological findings that are not supported by a clinical presentation, and thus reclassification of transient ischemic attacks to strokes is hardly possible.

An accurate assessment of trends requires constant quality of the register over the entire study period. In Kaunas, a city that served as one of the collaborating centers of the WHO MONICA project, a great effort was made to keep the same routine for case finding throughout the study, and the same diagnostic criteria were applied throughout the entire study period. This is the strength of our study. The upper age limit in our studied population was 65 years. This is a limitation of the present study in that it is unknown whether there are similar stroke trends in older people in whom the majority of events occur. Also, data on attack and incidence rates by stroke type should be treated with caution since these might be affected by an increasing use of imaging diagnostic techniques over the study period. In this study, we were not able to determine medication use in persons with strokes, and this is third limitation of the study.

In conclusion, during the period of 1986 to 2012, a flat trend in the incidence of stroke was observed middle-aged male and female population of Kaunas city, while attack rates were increasing due to the increase in recurrent strokes. Both mortality and 28-day case-fatality from stroke declined significantly over the study period in both sexes. An increase in the incidence and recurrence of stroke among middle-aged men and in the recurrence of stroke among women denotes inefficiency of measures used for both primary and secondary stroke prevention in Lithuania. A revision of the current prevention strategies and the introduction of new ones are of paramount importance in order to fight the epidemic of stroke.
